# 5-Amino­levulinic acid hydro­chloride

**DOI:** 10.1107/S2414314626004049

**Published:** 2026-04-29

**Authors:** Jean guillaume Ducreux, James A. Kaduk, Anja Dosen, Thomas N. Blanton

**Affiliations:** a North Central College, Department of Chemistry 131 S Loomis St Naperville IL 60540 USA; b ICDD, 12 Campus Blvd, Newtown Square, PA 19073, USA; Purdue University, USA

**Keywords:** powder diffraction, amino­levulinic acid, Rietveld refinement, density functional theory

## Abstract

The crystal structure of 5-amino­levulinic acid hydro­chloride (systematic name: 4-carb­oxy-2-oxobutan-1-aminium chloride), C_5_H_10_NO_3_^+^·Cl^−^, has been solved and refined using synchrotron X-ray powder diffraction data, and optimized using density functional theory (DFT) techniques. The structure is compared to a recent single-crystal determination [Wang *et al.* (2025 ▸). *Z. Kristallogr. New Cryst. Struct.***240**, 413–414]. 5-Amino­levulinic acid hydro­chloride crystallizes in the space group *Pbca* (No. 61), with *a* = 8.20862 (9), *b* = 11.22253 (10), *c* = 16.8595 (2) Å, *V* = 1553.12 (4) Å^3^, and *Z* = 8 at 298 K. The crystal structure consists of layers parallel to the *ab* plane. The center of the layers contains hydro­philic NH_3_, Cl, and CO_2_H groups, and the outer surface of the layers is composed of hydro­phobic CH_2_ groups. Hydrogen bonds are prominent in the crystal structure. The ammonium group acts as a donor to three chloride anions, and one hydrogen bond is bifurcated to the carbonyl group. Each Cl^−^ anion is an acceptor in three N—H⋯Cl hydrogen bonds, plus one from the carb­oxy­lic acid group. These hydrogen bonds connect the cations and anions into the layers parallel to the *ab* plane. The powder pattern has been submitted to the Inter­national Centre for Diffraction Data (ICDD) for inclusion in the Powder Diffraction File (PDF)

## Abstract 2

The crystal structure of 5-amino­levulinic acid hydro­chloride has been solved and refined using synchrotron X-ray powder diffraction data, and optimized using density functional theory techniques.

## Graphical abstract

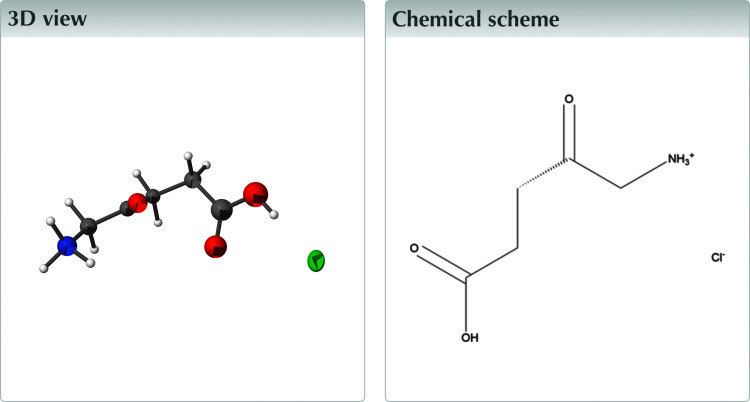

## Structure description

5-Amino­levulinic acid hydro­chloride is a porphyrin precursor and a photosensitizing agent. It finds application in the treatment of skin problems, including actinic keratosis and early-stage carcinomas. The systematic name (CAS Registry Number 5451-09-2) is 4-carb­oxy-2-oxobutan-1-aminium chloride. X-ray powder diffraction data for 5-amino­levulinic acid hydro­chloride has been reported in Chinese Patent CN113149854A (Gu *et al.*, 2021 ▸). The single-crystal structure of 5-amino­levulinic acid hydro­chloride has been determined very recently [Wang *et al.*, 2025 ▸; Cambridge Structural Database (CSD; Groom *et al.*, 2016 ▸) refcode MUPYOP]. This work is part of a project (Kaduk *et al.*, 2014 ▸) to determine commercial pharmaceutical crystal structures and add high-quality powder diffraction data to the Powder Diffraction File (Kabekkodu *et al.*, 2024 ▸).

**Figure 1:**
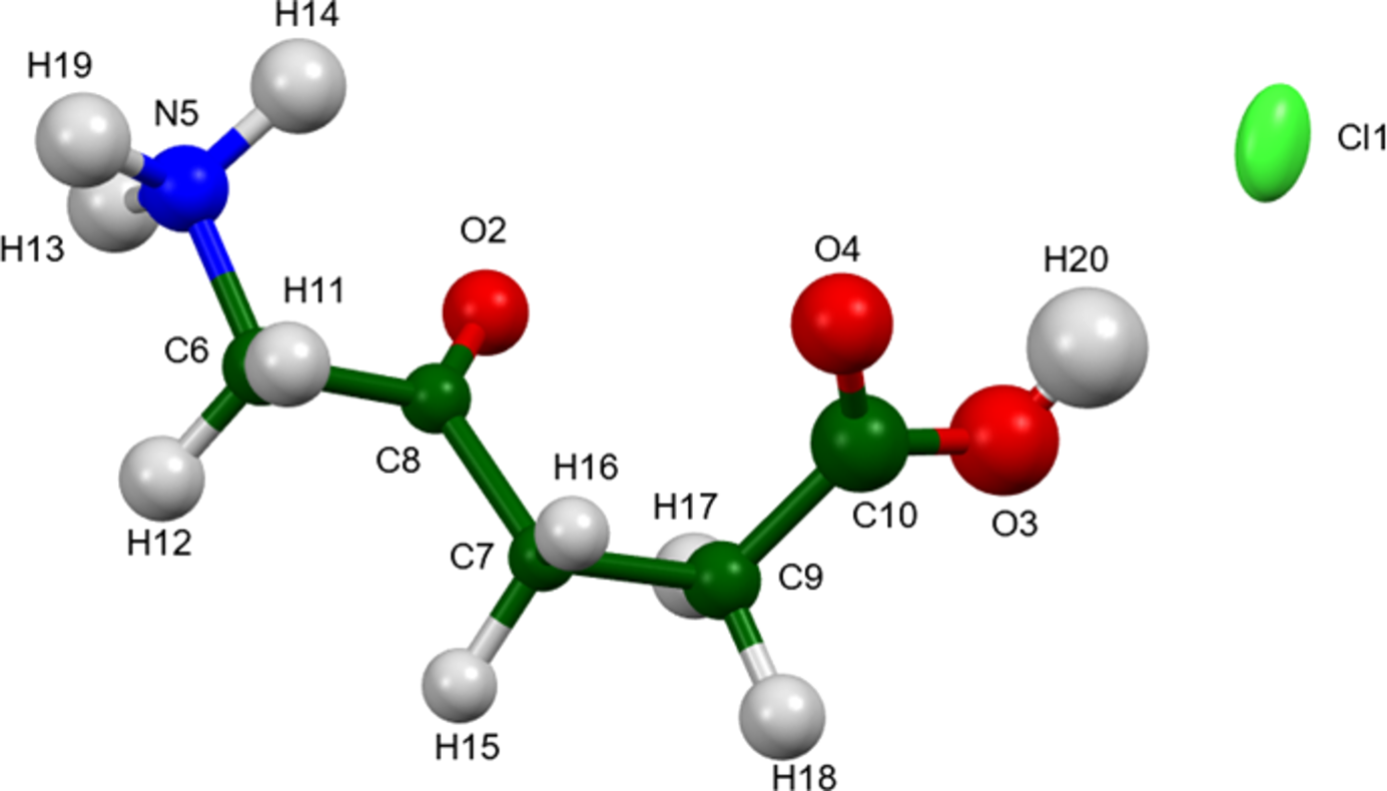
The asymmetric unit of 5-amino­levulinic acid hydro­chloride, with the atom numbering. The atoms are represented by 50% probability spheroids/ellipsoids. Image generated using *Mercury* (Macrae *et al.*, 2020 ▸).

The r.m.s. Cartesian displacement of the single-crystal (Wang *et al.*, 2025 ▸) and Rietveld-refined structures is 0.159 Å. The r.m.s. agreement of the Rietveld-refined and *VASP*-optimized structures is 0.172 Å. The agreement of the single-crystal, Rietveld, and *VASP* structures is very good. The agreements are all well within the normal range for correct structures (van de Streek & Neumann, 2014 ▸). The isotropic displacement coefficients from the Rietveld refinement correlate very well to the isotropic equivalents calculated form the anisotropic displacement coefficients from the single-crystal refinement, providing another measure of the accuracy of the powder structure. The standard uncertainties on the fractional coordinates from the Rietveld refinement average about 3.5 times larger than those from the single-crystal structure. The powder structure is accurate, but less precise, than the single-crystal structure. The asymmetric unit with the atom numbering is presented in Fig. 1 ▸.

All of the bond distances, bond angles, and torsion angles fall within the normal ranges indicated by a *Mercury Mogul* Geometry check (Macrae *et al.*, 2020 ▸). Quantum chemical geometry optimization of the isolated cation (DFT/B3LYP/6-31G*/water) using *Spartan ’24* (Wavefunction, 2023 ▸) indicated that it is 3.1 kcal mol^−1^ higher in energy than the local minimum, which has a similar conformation (r.m.s. displacement = 0.087 Å). The global minimum-energy conformation has the same energy, but a slightly different conformation (r.m.s. difference = 0.917 Å), mainly at the periphery of the cation. The cation is apparently flexible, and inter­molecular inter­actions determine the solid-state conformation.

**Figure 2:**
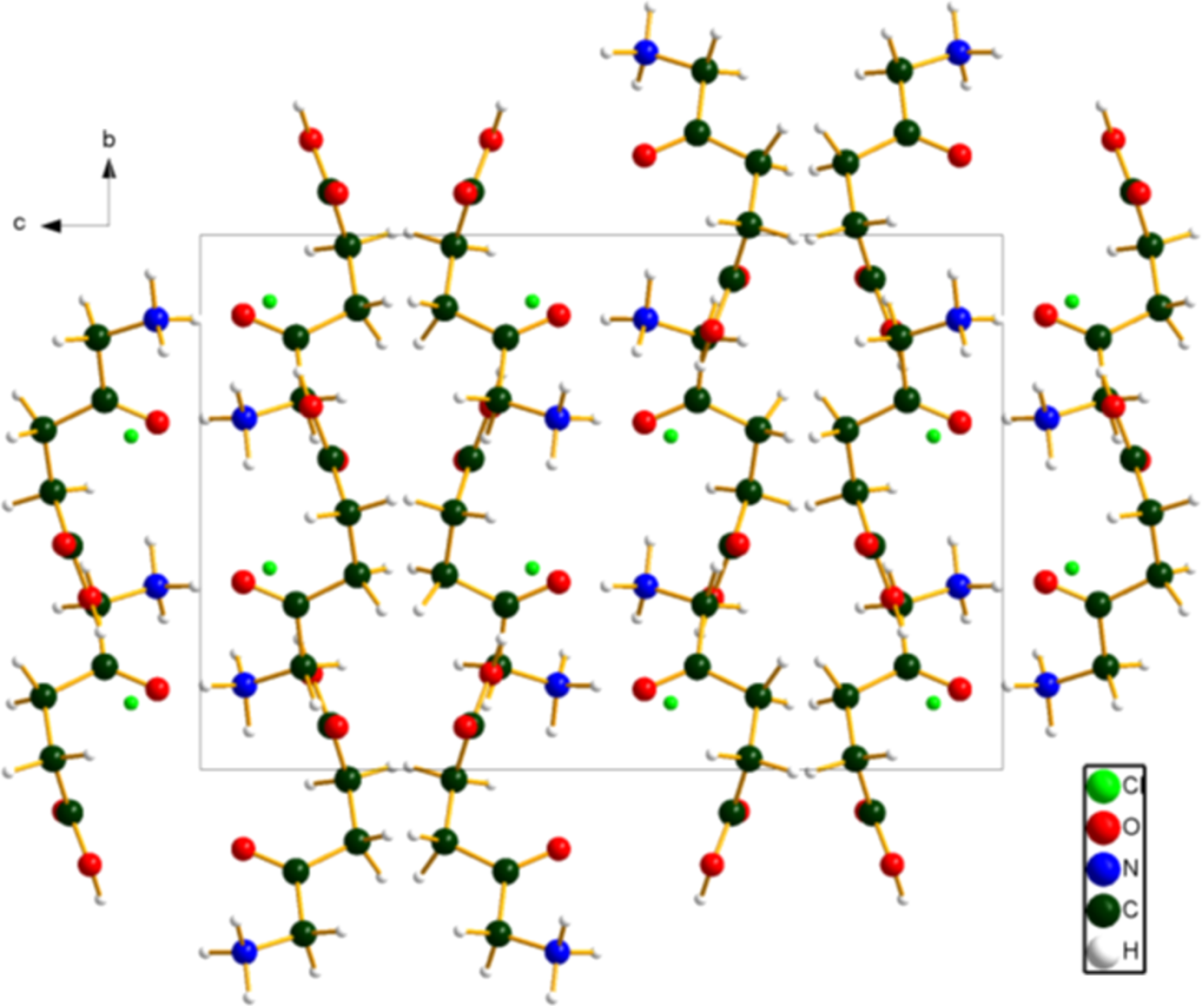
The crystal structure of 5-amino­levulinic acid hydro­chloride, viewed down the *a* axis. Image generated using *DIAMOND* (Putz & Brandenburg, 2025 ▸).

The crystal structure (Fig. 2 ▸) consists of layers parallel to the *ab* plane. The center of layers contains hydro­philic NH_3_, Cl, and CO_2_H groups, and the outer surface of the layers is composed of hydro­phobic CH_2_ groups.

Analysis of the contributions to the total crystal energy of the structure using the Forcite module of *Materials Studio* (Dassault Systèmes, 2024 ▸) suggests that the intra­molecular deformation energy is dominated by angle distortion terms, while van der Waals attractions (which in this force field-based analysis include hydrogen bonds) dominate the inter­molecular energy.

Hydrogen bonds are prominent in the crystal structure. The ammonium group acts as a donor to three chloride anions, and one hydrogen bond is bifurcated to the O2 carbonyl group. Each chloride anion is an acceptor in three N—H⋯Cl hydrogen bonds, plus one from the carb­oxy­lic acid group. These hydrogen bonds connect the cations and anions into layers parallel to the *ab* plane. The energies of the N—H⋯O hydrogen bonds were calculated using the correlation of Wheatley & Kaduk (2019 ▸), and the energy of the O—H⋯Cl hydrogen bond was calculated using the correlation of Kaduk (2002 ▸). Three C—H⋯O/Cl hydrogen bonds also contribute to the lattice energy.

The Bravais–Friedel–Donnay–Harker (Bravais, 1866 ▸; Friedel, 1907 ▸; Donnay & Harker, 1937 ▸) morphology suggests that we might expect isotropic morphology for 5-amino­levulinic acid hydro­chloride. A second-order spherical har­monic model was included in the refinement. The texture index was 1.010 (0), indicating that preferred orientation was not significant in this rotated capillary specimen.

## Synthesis and crystallization

5-Amino­levulinic acid hydro­chloride was a white powder purchased from TargetMol (Batch No. 146376), and was used as-received.

## Refinement

The powder sample was analyzed at 298 K at the Wiggler Low Energy Beamline (Leontowich *et al.*, 2021 ▸) of the Brockhouse X-ray Diffraction and Scattering Sector of the Canadian Light Source using a wavelength of 0.819325 (2) Å (15.1 keV). The pattern was indexed using *JADE Pro* (MDI, 2025 ▸) and the crystal structure was solved independently using direct methods, as implemented in *EXPO2014* (Altomare *et al.*, 2013 ▸). The original structure solution yielded the carb­oxy­lic acid group rotated ∼180° from the single-crystal structure. The single-crystal structure was 26.7 kcal/mol/cell lower in energy, and that conformation was adopted for the refinement.

**Figure 3:**
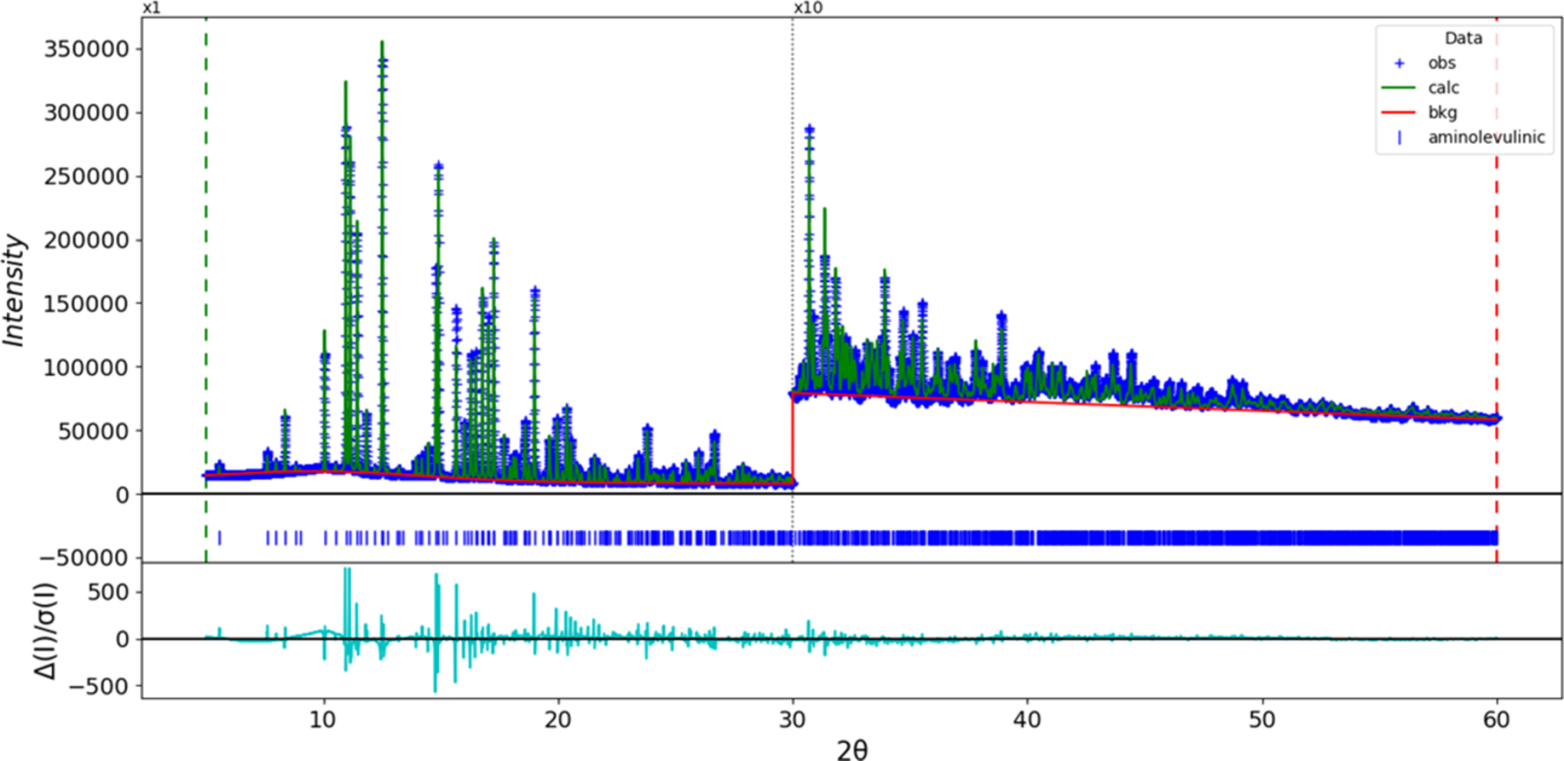
The Rietveld plot for 5-amino­levulinic acid hydro­chloride. The blue crosses represent the observed data points and the green line is the calculated pattern. The cyan curve is the normalized error plot and the red line is the background curve. The blue tick marks indicate the peak positions. The vertical scale has been multiplied by a factor of ×10 for 2θ > 30.0°.

Rietveld refinement (Fig. 3 ▸) was carried out using *GSAS-II* (Toby & Von Dreele, 2013 ▸). All non-H bond distances and angles were restrained according to a *Mercury/Mogul Geometry Check* (Sykes *et al.*, 2011 ▸; Bruno *et al.*, 2004 ▸). H atoms were included in calculated positions and recalculated during the refinement using the *Mercury* (Macrae *et al.*, 2020 ▸) ‘Auto Edit’ feature and the ‘Adjust Hydrogen’ feature of *Materials Studio* (Dassault Systèmes, 2024 ▸). The Cl atom was refined anisotropically. the *U*_iso_ values of the C, N, and O atoms were refined individually, while the *U*_iso_ values for the H atoms were fixed at 1.2 times the *U*_iso_ of the C, N, and O atoms to which they are attached. The final refinement yielded *R*_wp_ = 0.10874. The largest features in the normalized error plot are in the positions of many of the strong low-angle peaks, and may indicate that the specimen changed during the measurement. The largest peak (0.55 Å from Cl1) and hole (1.86 Å from Cl1) in the difference Fourier map were 0.87 (18) and −0.81 (18) e Å^−3^, respectively.

The crystal structure of 5-amino­levulinic acid hydro­chloride was optimized (fixed unit cell) with density functional theory (DFT) techniques using *VASP* (Version 6.0; Kresse & Furthmüller, 1996 ▸) through the *MedeA* graphical inter­face (Materials Design, 2024 ▸). Single-point density functional theory calculations (fixed experimental cell) and population analysis were carried out using *CRYSTAL23* (Erba *et al.*, 2023 ▸) using H, C, N, and O basis sets defined by Gatti *et al.* (1994 ▸) and the Cl basis set of Peintinger *et al.* (2013 ▸).

Experimental details are given in Table 1 ▸.

## Supplementary Material

Crystal structure: contains datablock(s) I. DOI: 10.1107/S2414314626004049/zl4095sup1.cif

The VASP-optimized structure and the hydrogen bonding. Necessary because IUCrData accommodates only one data block in the submission CIF. DOI: 10.1107/S2414314626004049/zl4095sup3.txt

CCDC references: 2548850, 2547145

Additional supporting information:  crystallographic information; 3D view; checkCIF report

Additional supporting information:  crystallographic information; 3D view; checkCIF report

## Figures and Tables

**Table 1:** Experimental details

Crystal data	
Chemical formula	C_5_H_10_NO_3_^+^·Cl^−^
*M*_r_	167.59
Crystal system, space group	Orthorhombic, *Pbca*
Temperature (K)	298
*a*, *b*, *c* (Å)	8.20862 (9), 11.22253 (10), 16.8595 (2)
*V* (Å^3^)	1553.12 (4)
*Z*	8
Radiation type	Synchrotron, λ = 0.81933 Å
μ (mm^−1^)	0.29
Specimen shape, size (mm)	Cylinder, 0.45 × 0.15

Data collection	
Diffractometer	Wiggler Low Energy Beamline, Brockhouse X-ray Diffraction and Scattering Sector, Canadian Light Source
Specimen mounting	Kapton capillary
Data collection mode	Transmission
Scan method	Step
2θ values (°)	2θ_min_ = −9.008 2θ_max_ = 75.047 2θ_step_ = 0.003

Refinement	
*R* factors and goodness of fit	*R*_p_ = 0.071, *R*_wp_ = 0.109, *R*_exp_ = 0.002, *R*(*F*^2^) = 0.09084, χ^2^ = 2566.942
No. of parameters	64
No. of restraints	34
(Δ/σ)_max_	7.079

Computer programs: *GSAS*-II (Toby & Von Dreele, 2013 ▸).

## full crystallographic data

### 4-Carboxy-2-oxobutan-1-aminium chloride Crystal data

**Table:**

C_5_H_10_NO_3_^+^·Cl^−^	*Z* = 8
*M**_r_* = 167.59	*D*_x_ = 1.434 Mg m^−^^3^
Orthorhombic, *P**b**c**a*	Synchrotron radiation, λ = 0.81933 Å
*a* = 8.20862 (9) Å	µ = 0.29 mm^−^^1^
*b* = 11.22253 (10) Å	*T* = 298 K
*c* = 16.8595 (2) Å	cylinder, 0.45 × 0.15 mm
*V* = 1553.12 (4) Å^3^	

### 4-Carboxy-2-oxobutan-1-aminium chloride Data collection

**Table:**

Wiggler Low Energy Beamline, Brockhouse X-ray Diffraction and Scattering Sector, Canadian Light Source diffractometer	Scan method: step
Specimen mounting: Kapton capillary	2θ_min_ = −9.008°, 2θ_max_ = 75.047°, 2θ_step_ = 0.003°
Data collection mode: transmission	

### 4-Carboxy-2-oxobutan-1-aminium chloride Refinement

**Table:**

Least-squares matrix: full	64 parameters
*R*_p_ = 0.071	34 restraints
*R*_wp_ = 0.109	0 constraints
*R*_exp_ = 0.002	Weighting scheme based on measured s.u.'s
*R*(*F*^2^) = 0.09084	(Δ/σ)_max_ = 7.079
33623 data points	Background function: Background function: "chebyschev-1" function with 3 terms: 7.73(3)e3, -1.90(5)e3, -8(3), Background peak parameters: pos, int, sig, gam: 9.68(4), 9.22(18)e6, 1.98(5)e5, 0.100,
Profile function: Finger-Cox-Jephcoat function parameters U, V, W, X, Y, SH/L: peak variance(Gauss) = Utan(Th)^2^+Vtan(Th)+W: peak HW(Lorentz) = X/cos(Th)+Ytan(Th); SH/L = S/L+H/L U, V, W in (centideg)^2^, X & Y in centideg 6.157, -1.198, 1.258, 0.000, 0.667, 0.002,	Preferred orientation correction: Simple spherical harmonic correction Order = 2 Coefficients: 0:0:C(2,0) = -0.185(6); 0:0:C(2,2) = -0.092(8)

### 4-Carboxy-2-oxobutan-1-aminium chloride Fractional atomic coordinates and isotropic or equivalent isotropic displacement parameters (Å^2^)

**Table:**

	*x*	*y*	*z*	*U*_iso_*/*U*_eq_	
Cl1	−0.08722 (19)	−0.38073 (14)	0.41224 (10)	0.0481	
O2	0.0446 (6)	0.1463 (3)	0.4452 (3)	0.0436 (19)*	
O3	0.1421 (6)	−0.1790 (3)	0.3666 (3)	0.072 (2)*	
O4	−0.0889 (6)	−0.0777 (4)	0.3361 (2)	0.061 (2)*	
N5	−0.1619 (6)	0.3359 (4)	0.4450 (3)	0.045 (2)*	
C6	−0.0760 (7)	0.3045 (5)	0.3692 (4)	0.036 (3)*	
C7	0.0829 (8)	0.1383 (5)	0.3041 (4)	0.028 (2)*	
C8	0.0181 (8)	0.1909 (6)	0.3815 (5)	0.029 (2)*	
C9	0.1717 (9)	0.0206 (6)	0.3188 (4)	0.037 (3)*	
C10	0.0637 (10)	−0.0816 (7)	0.3375 (4)	0.057 (3)*	
H11	−0.16952	0.29163	0.31995	0.0432*	
H12	0.01136	0.37928	0.35232	0.0432*	
H13	−0.07194	0.37918	0.48770	0.0535*	
H14	−0.21369	0.25158	0.47304	0.0535*	
H15	0.17071	0.20435	0.27562	0.0332*	
H16	−0.02297	0.12117	0.26185	0.0332*	
H17	0.26049	0.03343	0.37008	0.0438*	
H18	0.24389	−0.00500	0.26360	0.0438*	
H19	−0.26527	0.40077	0.43180	0.0535*	
H20	0.07050	−0.24190	0.38070	0.0865*	

### 4-Carboxy-2-oxobutan-1-aminium chloride Atomic displacement parameters (Å^2^)

**Table:**

	*U*^11^	*U*^22^	*U*^33^	*U*^12^	*U*^13^	*U*^23^
Cl1	0.0527 (18)	0.0384 (15)	0.053 (2)	0.006 (3)	−0.026 (3)	−0.018 (3)

### 4-Carboxy-2-oxobutan-1-aminium chloride Geometric parameters (Å, º)

**Table:**

O2—C8	1.205 (8)	C8—C7	1.528 (8)
O3—C10	1.360 (7)	C9—C7	1.529 (7)
O3—H20	0.949 (4)	C9—C10	1.483 (8)
O4—C10	1.254 (8)	C9—H17	1.140 (6)
N5—C6	1.502 (8)	C9—H18	1.141 (7)
N5—H13	1.139 (5)	C10—O3	1.360 (7)
N5—H14	1.140 (5)	C10—O4	1.254 (8)
N5—H19	1.140 (5)	C10—C9	1.483 (8)
C6—N5	1.502 (8)	H11—C6	1.140 (6)
C6—C8	1.505 (6)	H12—C6	1.140 (6)
C6—H11	1.140 (6)	H13—N5	1.139 (5)
C6—H12	1.140 (6)	H14—N5	1.140 (5)
C7—C8	1.528 (8)	H15—C7	1.140 (6)
C7—C9	1.529 (7)	H16—C7	1.140 (6)
C7—H15	1.140 (6)	H17—C9	1.140 (6)
C7—H16	1.140 (6)	H18—C9	1.141 (7)
C8—O2	1.205 (8)	H19—N5	1.140 (5)
C8—C6	1.505 (6)	H20—O3	0.957 (4)
			
C10—O3—H20	113.3 (5)	C8—C7—H16	109.5 (6)
C6—N5—H13	109.5 (5)	C9—C7—H16	108.6 (5)
C6—N5—H14	109.4 (4)	H15—C7—H16	109.2 (5)
H13—N5—H14	109.5 (5)	O2—C8—C6	124.5 (8)
C6—N5—H19	109.4 (5)	O2—C8—C7	122.6 (7)
H13—N5—H19	109.5 (4)	C6—C8—C7	112.9 (7)
H14—N5—H19	109.5 (4)	C7—C9—C10	114.7 (6)
N5—C6—C8	108.8 (6)	C7—C9—H17	108.6 (5)
N5—C6—H11	109.5 (5)	C10—C9—H17	108.7 (6)
C8—C6—H11	109.8 (5)	C7—C9—H18	109.4 (6)
N5—C6—H12	109.5 (6)	C10—C9—H18	106.8 (5)
C8—C6—H12	109.6 (5)	H17—C9—H18	108.6 (6)
H11—C6—H12	109.6 (6)	O3—C10—O4	120.5 (8)
C8—C7—C9	111.1 (6)	O3—C10—C9	114.5 (7)
C8—C7—H15	109.1 (5)	O4—C10—C9	124.5 (7)
C9—C7—H15	109.2 (6)		
